# Deep vector-based convolutional neural network approach for automatic recognition of colonies of induced pluripotent stem cells

**DOI:** 10.1371/journal.pone.0189974

**Published:** 2017-12-27

**Authors:** Muthu Subash Kavitha, Takio Kurita, Soon-Yong Park, Sung-Il Chien, Jae-Sung Bae, Byeong-Cheol Ahn

**Affiliations:** 1 Department of Nuclear Medicine, Kyungpook National University, School of Medicine and Hospital, Daegu, Korea; 2 Graduate School of Engineering, Hiroshima University, Hiroshima, Japan; 3 School of Computer Science and Engineering, Kyungpook National University, Daegu, Korea; 4 School of Electronics Engineering, Kyungpook National University, Daegu, Korea; 5 Department of Physiology, School of Medicine, Kyungpook National University, Daegu, Korea; University of South Carolina, UNITED STATES

## Abstract

Pluripotent stem cells can potentially be used in clinical applications as a model for studying disease progress. This tracking of disease-causing events in cells requires constant assessment of the quality of stem cells. Existing approaches are inadequate for robust and automated differentiation of stem cell colonies. In this study, we developed a new model of vector–based convolutional neural network (V-CNN) with respect to extracted features of the induced pluripotent stem cell (iPSC) colony for distinguishing colony characteristics. A transfer function from the feature vectors to the virtual image was generated at the front of the CNN in order for classification of feature vectors of healthy and unhealthy colonies. The robustness of the proposed V-CNN model in distinguishing colonies was compared with that of the competitive support vector machine (SVM) classifier based on morphological, textural, and combined features. Additionally, five-fold cross-validation was used to investigate the performance of the V-CNN model. The precision, recall, and *F*-measure values of the V-CNN model were comparatively higher than those of the SVM classifier, with a range of 87–93%, indicating fewer false positives and false negative rates. Furthermore, for determining the quality of colonies, the V-CNN model showed higher accuracy values based on morphological (95.5%), textural (91.0%), and combined (93.2%) features than those estimated with the SVM classifier (86.7, 83.3, and 83.4%, respectively). Similarly, the accuracy of the feature sets using five-fold cross-validation was above 90% for the V-CNN model, whereas that yielded by the SVM model was in the range of 75–77%. We thus concluded that the proposed V-CNN model outperforms the conventional SVM classifier, which strongly suggests that it as a reliable framework for robust colony classification of iPSCs. It can also serve as a cost-effective quality recognition tool during culture and other experimental procedures.

## Introduction

Induced pluripotent stem cells (iPSCs), which are created from an adult cell that has been reprogrammed, enable the development of an unlimited source of any type of human cells needed for drug discovery and clinical applications [1]. iPSCs are able to help track the earliest disease-causing events in cells and can be used as sources of various cell-based therapies. Because a healthy quality of undifferentiated iPSCs is an essential requisite for further experimental and therapeutic approaches, the rapid and robust estimation of iPSC quality is very important to meet growing demands [2–4]. The morphological structure of a healthy or good-quality iPSC colony commonly has tightly compacted round cells and an explicit boundary, whereas unhealthy or bad-quality colonies show a different morphology [5]. The present approach of evaluating the quality of iPSCs on the basis of colony morphology is predominantly subjective and can strongly differ according to individual skills. Therefore, a quantitative system for the rapid and accurate segmentation and estimation of colony quality is essential in order to reduce classification errors. Furthermore, removal of the use of fluorescent labeling or other chemical reagents would be helpful in preparing the iPSCs for additional research experiments.

Automated segmentation of stem cell colonies for phase contrast imaging is challenging and requires specialized algorithms to handle the problems of halo artifacts and overlapping of the colony edges with the feeder cells [6]. The currently available image analysis techniques to achieve stem cell colony selection are based on morphological operations, thresholding, and watershed transformation. A combination of these techniques is designed to examine the status of the colony in each individual research [7–9]. Alternatively, other approaches have adopted commercial software tools that basically use filtering, automatic thresholding, and Voronoi algorithms for stem cell segmentation and tracking [1, 5, 10, 11]. In addition, the morphological categories of colonies based on commercial program require manual interpretation to locate the colony area for feature measurement [5]. Because these aforementioned image analysis techniques are quite problem specific and rely strictly on parametric settings, they lack controllability to manipulate variations among the stem cell heterogeneity on a large scale.

Recently, several supervised machine learning approaches have also been developed and their significance in distinguishing stem cell colonies confirmed [12–15]. The approaches designed for the selection of colonies, based on *k*-nearest neighbor searching with [13] and without error correction output codes [14], ensemble support vector machine (SVM) [12], and random forest methods [15], acquired local features from the patches of original images. In addition, the development of the learning set for discrimination of cells in the colony has to be done manually. However, a high degree of reliability and cost-effectiveness is very important in clinical applications. Recently, many researchers have focused on implementing the convolutional neural network (CNN) for various medical imaging modalities, and its high reliability and validity for object segmentation and detection applications have been revealed [16, 17]. In addition, CNNs have been successfully applied to microscopic cell imaging data, with robust decisions on ambiguous cell classifications found, making the process suitable for clinical applications [18–20]. A deep CNN method for identifying mitosis in the cell nucleus reportedly had higher satisfactory performance than estimation with conventional methods [21]. A deep multiple instance learning-based CNN approach effectively segmented mammalian and yeast microscopy images with remarkable accuracy [18]. However, the above-mentioned CNN approaches on biomedical imaging data differ from our study, as they have adopted deep neural networks for image segmentation tasks. In this study, our target was to train a CNN model to classify colonies on the basis of obtained features of the segmented colony. Most of the aforementioned studies tested colony morphology for estimating colony categories. However, apart from certain quantitative morphological features, textural features are the most important, as they describe the spatial intensity variations of the colony image. Furthermore, textural features are closely connected with cellular characteristics [22]. Hence, this study considered both colony morphological and textural features for the evaluation of iPSCs.

The objectives of this study were 1) to determine, whether or not the proposed feature vector-based convolutional neural network (V-CNN) is the most suitable and best model of colony quality recognition from the morphological and textural features of a segmented colony; 2) to confirm the promising results of colony quality recognition through use of an accurate cross-validation process; 3) to demonstrate the superiority of the proposed deep V-CNN learning approach over the SVM classification system.

## Materials and methods

### Cell culture and image data acquisition

The iPSCs were maintained as described previously [23]. For inactive murine embryonic fibroblasts (MEFs) isolation, we used day 13.5 embryos. After the removal of the head, visceral tissues, and gonads, the remaining bodies were washed and dissociated with 0.25% trypsin-EDTA (Sigma-Aldrich, Saint Louis, MI, USA). Ten-million cells were plated on each gelatin-coated 100- mm dish and incubated at 37°C with 5% CO2. The next day, floating cells were removed by washing with PBS. In this study, MEFs were used within passage 4 to avoid replicative senescence. Normal iPSC line (HPS0063) was obtained from the RIKEN Bioresource Center [24]. The harvested colonies were triturated to generate medium-sized small fragments, which were then seeded on new plates together with the mitomycin C-treated MEFs in complete ES medium composed of DMEM (Sigma-Aldrich) supplemented with 20% knockout serum replacement, 5 ng ml^−1^ recombinant human basic fibroblast growth factor (Peprotech), 20 mM HEPES buffer (pH 7.3), 0.1 mM 2-mercaptoethanol, 0.1 mM non-essential amino acids, 2 mM l-glutamine and 100 U ml^−1^ penicillin/streptomycin (all other materials were from Gibco). All images were prepared under the 100× objective of the phase contrast microscope in the BioStation CT system, using automatic Z-focus with a resolution of 1360 × 1024 pixels.

In addition this study analyzes the variations of marker expression among iPSC samples using TRA-1-60 and TRA-1-81 antibody (mouse, 1:100, Chemicon, Billerica, MA, http://www.chemicon.com/). The cells were analysed with a laser scanning confocal microscope equipped with Fluoview SV1000 imaging software (Olympus FV1000, Olympus, Tokyo, Japan). It expressed only on the established healthy iPSCs and not on unhealthy iPSCs which is presented in S1 Fig. Hence the variations of marker expression among healthy and unhealthy colonies and morphology were used to label the iPSC samples used in this study.

### iPSC colony segmentation

**Fig 1:**
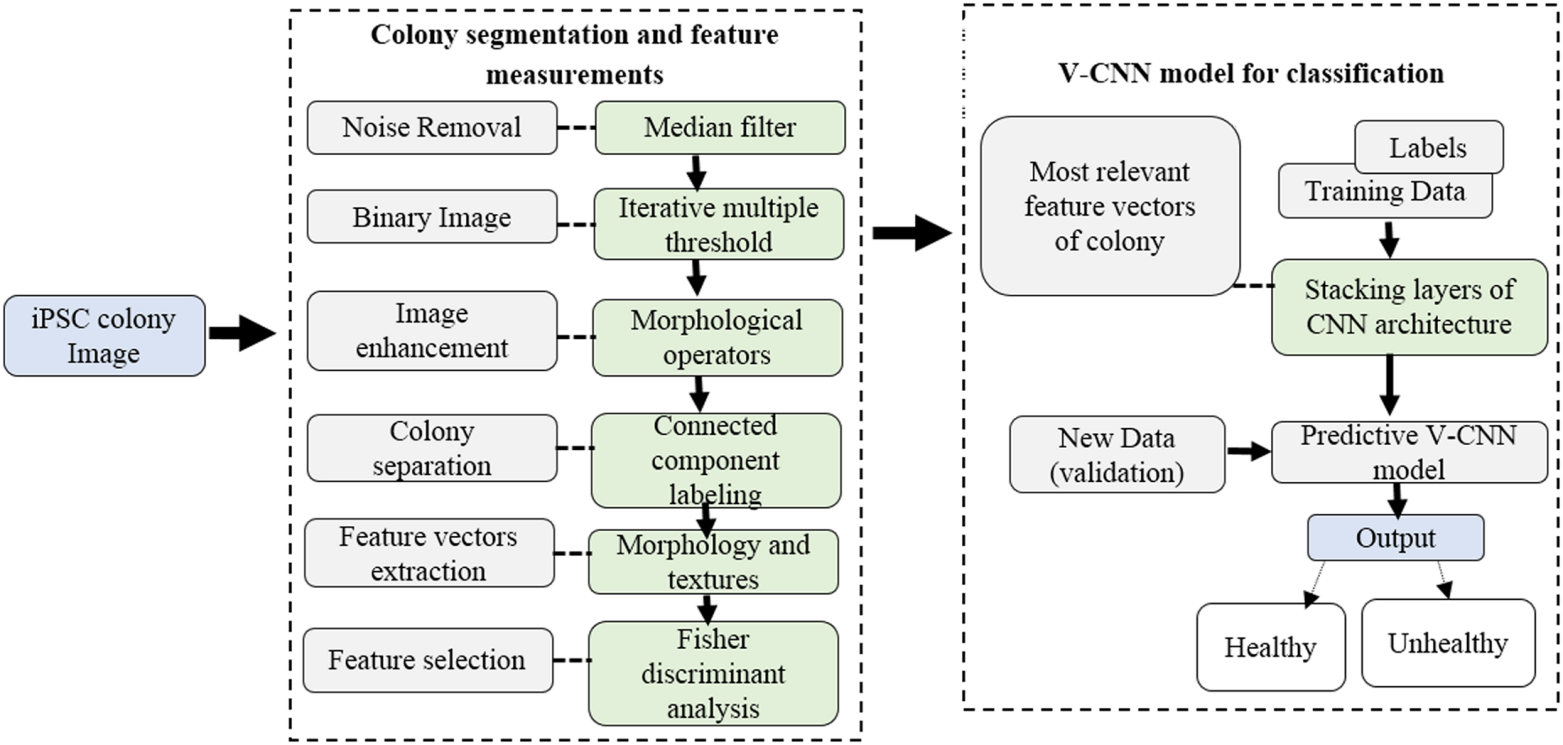
Block diagram of the proposed deep vector-based convolutional neural network for classification of induced pluripotent stem cell colonies.

A block diagram of the proposed automated system is shown in Fig 1. As mentioned above, the system interfaces image analysis methods with the V-CNN model for segmenting colonies in order to compute their morphological and textural features for use in their classification by deep learning architecture. Robust segmentation of the colony region prior to classification is beneficial for automating pluripotency. However, the computerized segmentation of colony regions with feeder cells included is more challenging for the subsequent measurement of stem cell characteristics [25]. In this study, the entire colony image was used for the segmentation of the colony region. In the beginning of the process, median filtering was used as a preprocessing step to reduce the background noise and further to preserve the edges of the stem cell regions. This works on the original image object, replacing the center value of the window with the median value of all neighboring pixel values. A median mask size value of 9 × 9 pixels was applied to the original image.

After the preprocessing step, we used an iterative multiple thresholding algorithm to separate the image pixels into the foreground and background, where the threshold estimation depends on maximization of the between-class variances of the pixel values [26]. This estimates the thresholds iteratively and returns two optimal thresholds. The iteration continues until the errors become small or the thresholds no longer change. We initialized the thresholds *t*_1_ and *t*_2_ as *I*/3 and 2*I*/3, respectively, where *I* indicates the intensity range of the image. The error functions are represented as

$$\varepsilon_{{}_{1}}(t_{{}_{1}},t_{{}_{2}})=\left[m(0,t_{1})+m(t_{1},t_{2})\right]/2-t_{1}$$ (1)

and

$$\varepsilon_{2}(t_{1},t_{2})=\left[m(t_{1},t_{2})+m(t_{2},\infty )\right]/2-t_{2}$$ (2)

where

$$m(t_{m},t_{n})=\frac{1}{t_{{}_{m}}+t_{n}+1}\sum_{h=t_{m}}^{t_{n}}w(h).h$$ (3)

where *w*(*h*) indicates the image histogram. The thresholds *t*_1_ and *t*_2_ were updated to force the errors *ε*_1_ and *ε*_2_ toward zero. The updated thresholds are represented as

$$t_{1}^{\prime }\;=t_{1}+\varepsilon_{1}$$ (4)

$$t_{2}^{\prime }\;=t_{2}+\varepsilon_{2}$$ (5)

**Fig 2:**
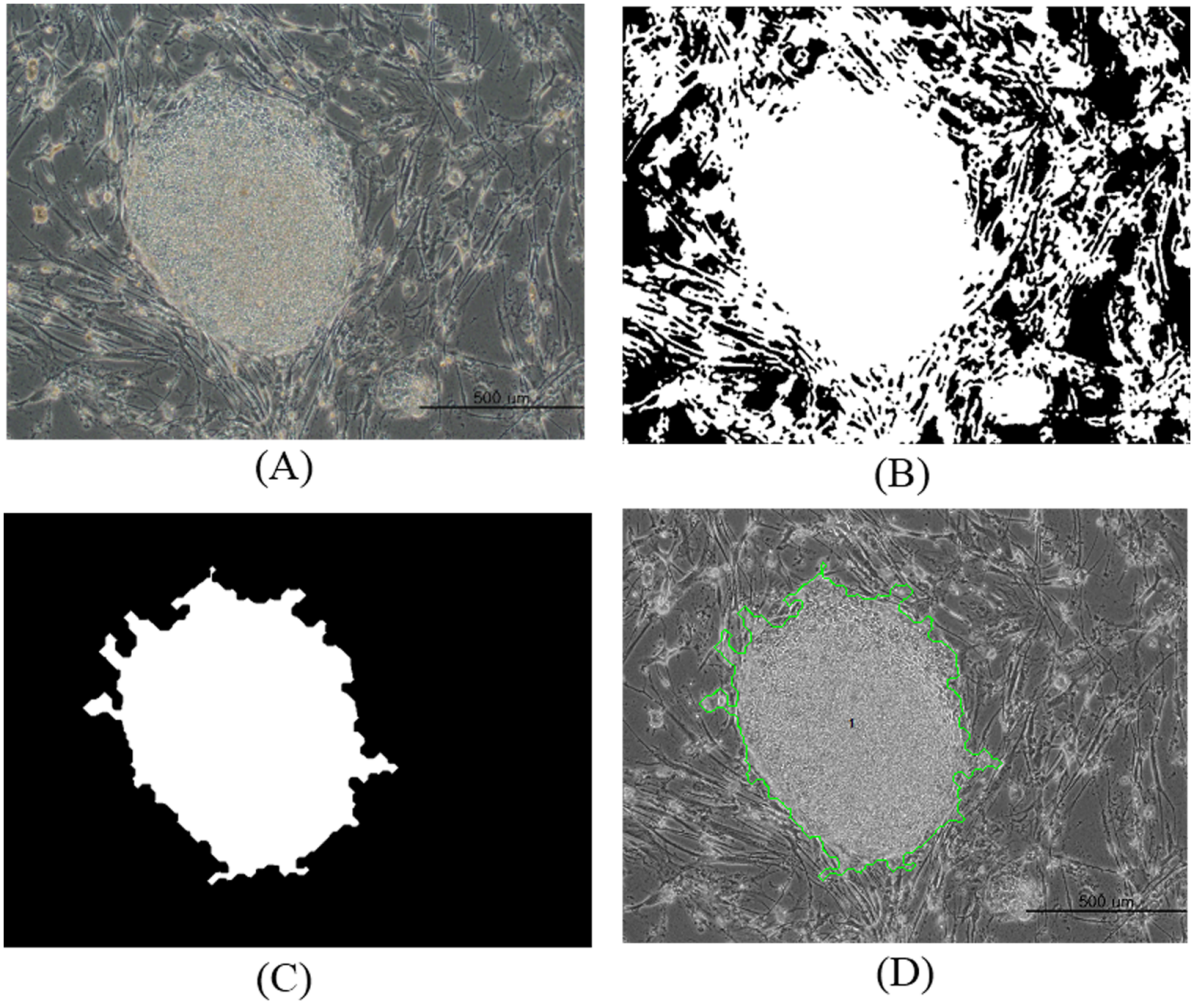
Quantitative feature measurements for a healthy image of a segmented induced pluripotent stem cell colony. (A) Original image. (B) Iterative thresholding. (C) Morphological operation with size filter (D) Labeling.

**Fig 3:**
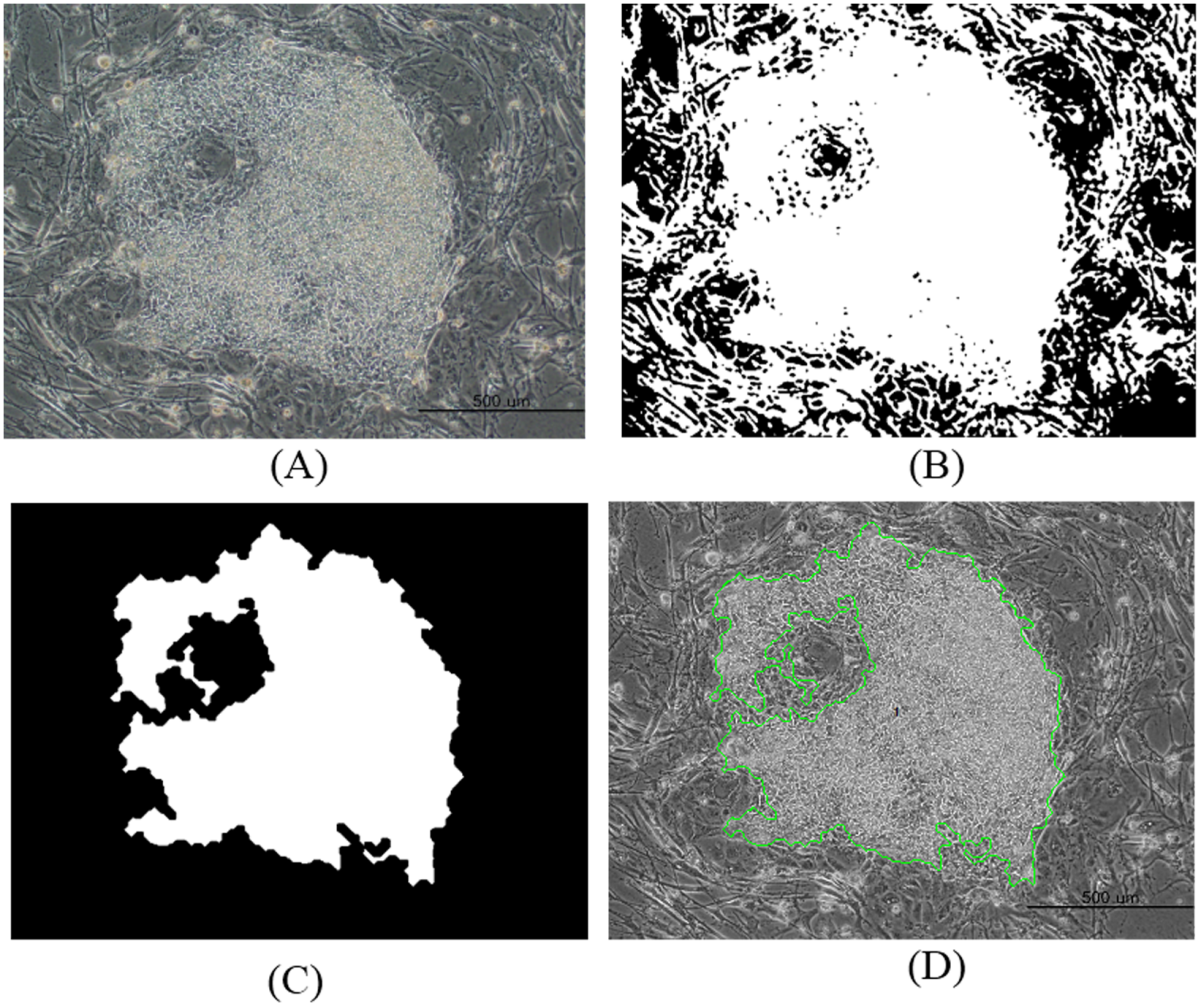
Quantitative feature measurements for an unhealthy image of a segmented induced pluripotent stem cell colony. (A) Original image. (B) Iterative thresholding. (C) Morphological operation with size filter (D) Labeling.

The resultant binary image was then processed using morphological closing and opening operations. It was closed using a disk-shaped structuring element with a radius of 2, and opened using a diamond-shaped structuring element with a distance of 19. The resultant connected components were then filled, and the contours of the objects were smoothed with morphological erosion and hole-filling operations. Furthermore, the unwanted cells around the colony regions, which are smaller than the user-specified threshold, were removed using a size filtering method. We noticed that a size of 9000 pixels was suitable for removing the other regions that surround the colony area. Finally, the resultant segmented colony region was evaluated for further quantitative feature measurements by connected component labeling with eight-neighbor connectivity. This method is generally used to estimate adjacent pixels that share the same set of intensity values [27]. The segmentation results of the healthy and unhealthy colony image are shown in Figs 2 and 3, respectively.

### Colony feature extraction and selection

**Fig 4:**
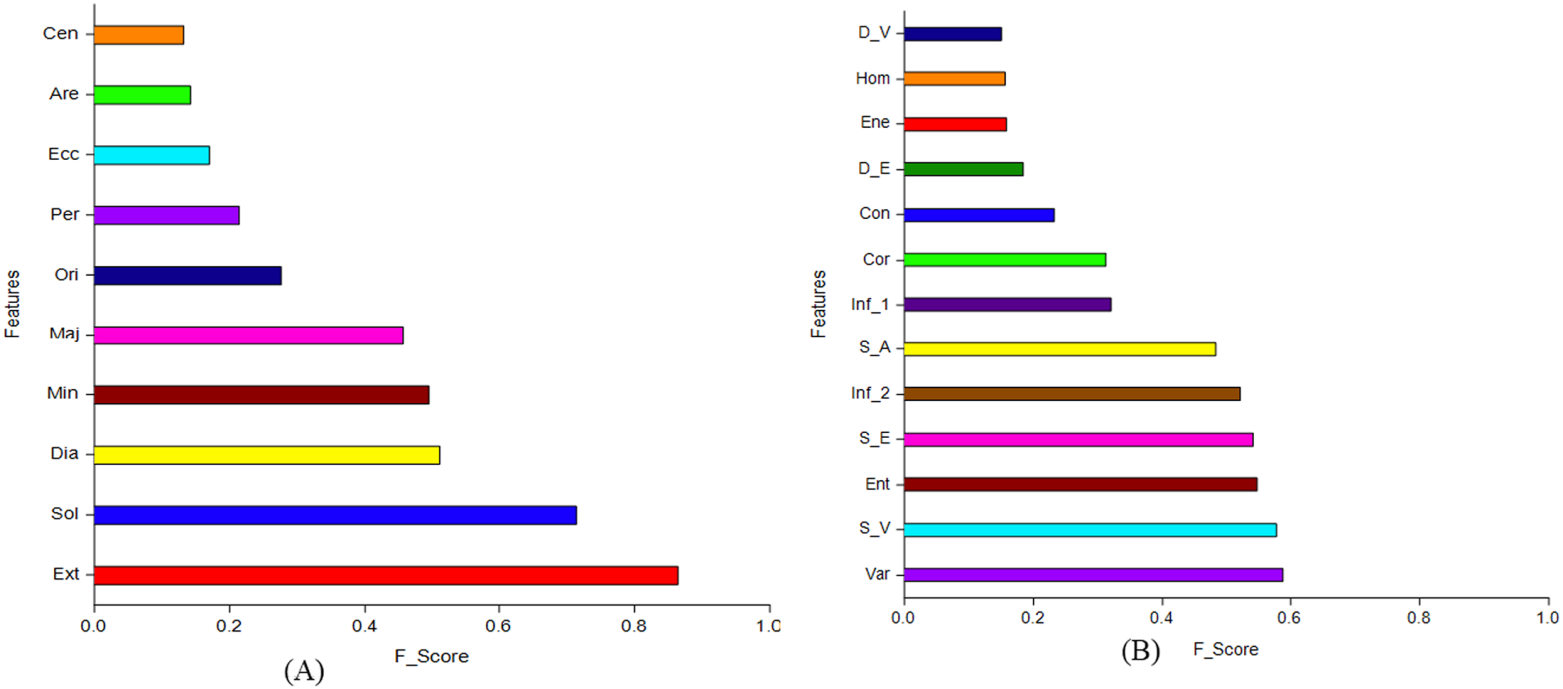
Fisher scores assigned to each feature in the feature sets of (A) morphology, and (B) textures. Cen, centroid; Are, area; Ecc, eccentricity; Per, perimeter; Ori, orientation; Maj, major axis; Min, minor axis; Dia, equivalent diameter; Sol, solidity; Ext, extent; D_V, difference variance; Hom, homogeneity; Ene, energy; D_E, difference entropy; Con, contrast; Cor, correlation; Inf_1, information measure of correlation_1; S_A, sum average; Inf_2, information measure of correlation_2; S_E, sum entropy; Ent, entropy; S_V, sum variance; Var, variance.

Each colony region was estimated for ten morphological features; namely, area, perimeter, centroid, equivalent diameter, eccentricity, solidity, major axis, minor axis, extent, and orientation. The definitions of these features are described in S1 Table. The textural features adopted in this study can be explained in terms of a gray-level co-occurrence matrix of 13 features (for details, see [28]), which reveals the different combinations of pixel intensity values in a specific spatial displacement. The most relevant features of colony categories are identified using the feature selection technique. In this study, Fisher score analysis was applied to determine the most relevant features for the subsequent classification task, while excluding the irrelevant ones. Fisher scores were automatically computed for each feature in the feature sets of training data (Fig 4) and used to select the informative features by which the within-class distance is minimized and the between-class distance is maximized [29]. Specifically, given the selected *f* features, the input data matrix *X* ∊ *R*^*a*×*n*^ reduces to *Q* ∊ *R*^*m*×*n*^. Hence, the Fisher score is represented as follows:

$$\underset{Q}{\text{arg}\;\text{max}\;tr}\;\;\left\{{\tilde{V}}_{t}{}^{-1}{\tilde{V}}_{b}\right\}$$ (6)

where ${\tilde{V}}_{t}$ and ${\tilde{V}}_{b\;}$ are defined as

$${\tilde{V}}_{t}=\sum_{i=1}^{n}(Q_{i}-\tilde{\mu })\;{\left(Q_{i}-\tilde{\mu }\right)}^{L},\;{\tilde{V}}_{b}=\sum_{k=1}^{c}n_{k}({\tilde{\mu }}_{k}-\tilde{\mu })\;{\left({\tilde{\mu }}_{k}-\tilde{\mu }\right)}^{L}$$ (7)

where ${\tilde{\mu }}_{k}$ and *n*_*k*_ are the mean vector and size of the *k*^*th*^ class, respectively, in the reduced data space; that is, $Q\;\tilde{\mu }=\sum_{k=1}^{c}n_{k}{\tilde{\mu }}_{k}$ is the overall mean vector of the reduced data.

### Vector-based convolutional neural network for classification

**Fig 5:**
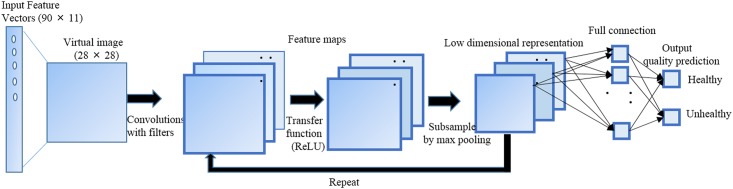
V-CNN architecture for recognition of induced pluripotent stem cell colony quality.

CNNs are a branch of neural networks that have been implemented successfully in image recognition and classification [16–21]. Although the CNN has been applied for various medical imaging segmentations, it has not been used previously for the input of feature vector-based classifications for colony quality. The selected features of colony morphology and textures obtained from the segmented colony were entered into the V-CNN model in order for the classification task to identify the colony quality. However, input feature vectors cannot be entered directly into the typical CNN. Hence, we added a transfer function from the feature vectors to the virtual image at the front of the CNN model organization. In addition, the parameters of the mapping function were trained to obtain an adequate transfer function for a target classification task of the CNN framework. Hereafter, we briefly explain the mathematical framework of V-CNN and the process of training to implement the classification task. The V-CNN architecture used in this study is arranged by stacking a set of convolutional, transfer function, and pooling layers in an alternate way, as shown in Fig 5. The main work of the convolutional layer is to estimate local conjunctions of features from the input feature vectors and to map their occurrence to a feature map. As a result of convolution in neuronal networks, the feature vectors are partitioned into perceptrons, generating local flexible fields and finally trampling the perceptron into feature maps of size *n*_1_ × *n*_2_. In each layer, there is a bank of *n* filters that detect features at every location of the input. The output $Y_{a}^{\left(q\right)}$ of layer *q* consists of *n*^(*q*)^ feature maps of size $n_{1}^{\left(q\right)}\;\times \;n_{2}^{\left(q\right)}$. The *a*^*th*^ feature map, indicated as $Y_{a}^{\left(q\right)}$, is computed as

$$Y_{a}^{\left(q\;\right)}\;=G_{a}^{\left(q\right)}\;+\;\sum_{b=1}^{n^{\left(q-1\right)}}V_{a,b}^{\left(q\right)}*\;Y_{b}^{\left(q-1\right)}$$ (8)

where $G_{a}^{\left(q\right)}$ is a bias matrix and $V_{a,b}^{\left(q\right)}$ is the filter of size $2t_{1}^{\left(q\right)}+1\times 2t_{2}{}^{\left(q\right)}+1$, connecting the *b*^*th*^ feature map in layer (*q* − 1) with the *a*^*th*^ feature map in the layer. The weights of these filters and their values are altered throughout the training to reduce the classification error on any training data. The next operation is to apply the transfer function to produce a set of feature maps. This helps the classifier to build nonlinear decision boundaries. The selection of an activation function has a strong influence on the computational costs of both training and validation performances. Hence, in this study, we chose the rectified linear unit (ReLU) as a transfer function, which is many times faster than the other activation functions. It is defined as

$$Y_{a}^{\left(q\right)}\;=\text{max}(0,Y_{a}^{\left(q-1\right)})$$ (9)

The third operation is the max-pooling layer, which partitions the input feature vectors into a set of non-overlapping rectangles and returns the maximum value of each such rectangular feature set. Furthermore, it significantly reduces the input size and number of network parameters, hence controlling CNN overfitting. It is usually implemented after multiple stages of convolutional and nonlinearity layers in order to minimize both the computational requirements as well as the likelihood of overfitting. The max-pooling layer *q* has two hyper parameters: the spatial extent of the filter *F*^(*q*)^, and the step size *S*^(*q*)^. The pooling layer describes a window of size *F*^(*q*)^ × *F*^(*q*)^ and minimizes the data within this window to a single value. Similarly to the convolutional layer, the window is moved by *S*^(*q*)^ positions after each operation. The minimization of the data is repeated at each position of the window until the entire activation volume is spatially reduced. In this study, we evaluated max pooling with a 2 × 2 window, using a step size of 2. The output feature maps of these operations can then be fed as the input to another round of the same three operations (convolutional, ReLU, max-pooling layers). The last operation for final classification is to create fully connected layers of mono-dimensional features, which are designed to map the activation volume from the fusion of previous different layers to a class probability distribution. If (*q* − 1) is a fully connected layer, then it is defined as

$$y_{a}^{\left(q\right)}\;=\;f(z_{a}^{\left(q\right)})$$ (10)

where

$$\;z_{a}^{\left(q\right)}\;=\sum_{b=1}^{n^{\left(q-1\right)}}w_{a,b}^{\left(q\right)}y_{a}^{\left(q-1\right)}$$ (11)

The purpose of the complete fully connected structure is to tune the weight parameters $\;w_{a,b}^{\left(q\right)}$ to produce a stochastic likelihood representation of each class found on the activation maps created by the combination of convolutional, ReLU, and pooling layers. The repeated implementation of these two operations generates an output vector of class scores, which assists as the classification prediction. In addition, a cost function is employed to reduce the classification error. In this study, we applied a soft-max cost function in order to generate a probability output in the range of 0 to 1 that can automatically be converted to class values. We implemented training and testing of the V-CNN in Python, using the Keras, TensorFlow, NumPy, SciPy, and Scikit-learn Python packages [30–33]. The training data were partitioned into fixed batch sizes (10) of the input feature vectors. All the batches of input feature vectors were evaluated in 20 epochs, which means that the procedure ran 20 times on the entire training data set. The V-CNN was trained with the TensorFlow framework at a learning rate of 0.001, using the Adam optimizer for cross-entropy minimization. The scale of the samples were evaluated using train_test_split () function and random_state parameter with different seed values to ensure the reproducibility of the classification performance of the model. The accuracy and loss values were evaluated to show the fitness of the model. The performance of the proposed V-CNN model for classifying the colony quality was estimated using the morphological feature, textural feature, and combined morphological and textural features (hereafter referred simply to as “combined features”).

## Results

**Fig 6:**
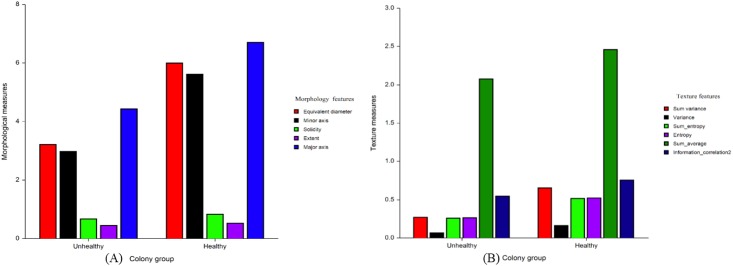
Comparison of the ranges of values between healthy and unhealthy colony groups based on (A) morphological and (B) textural features.

**Fig 7:**
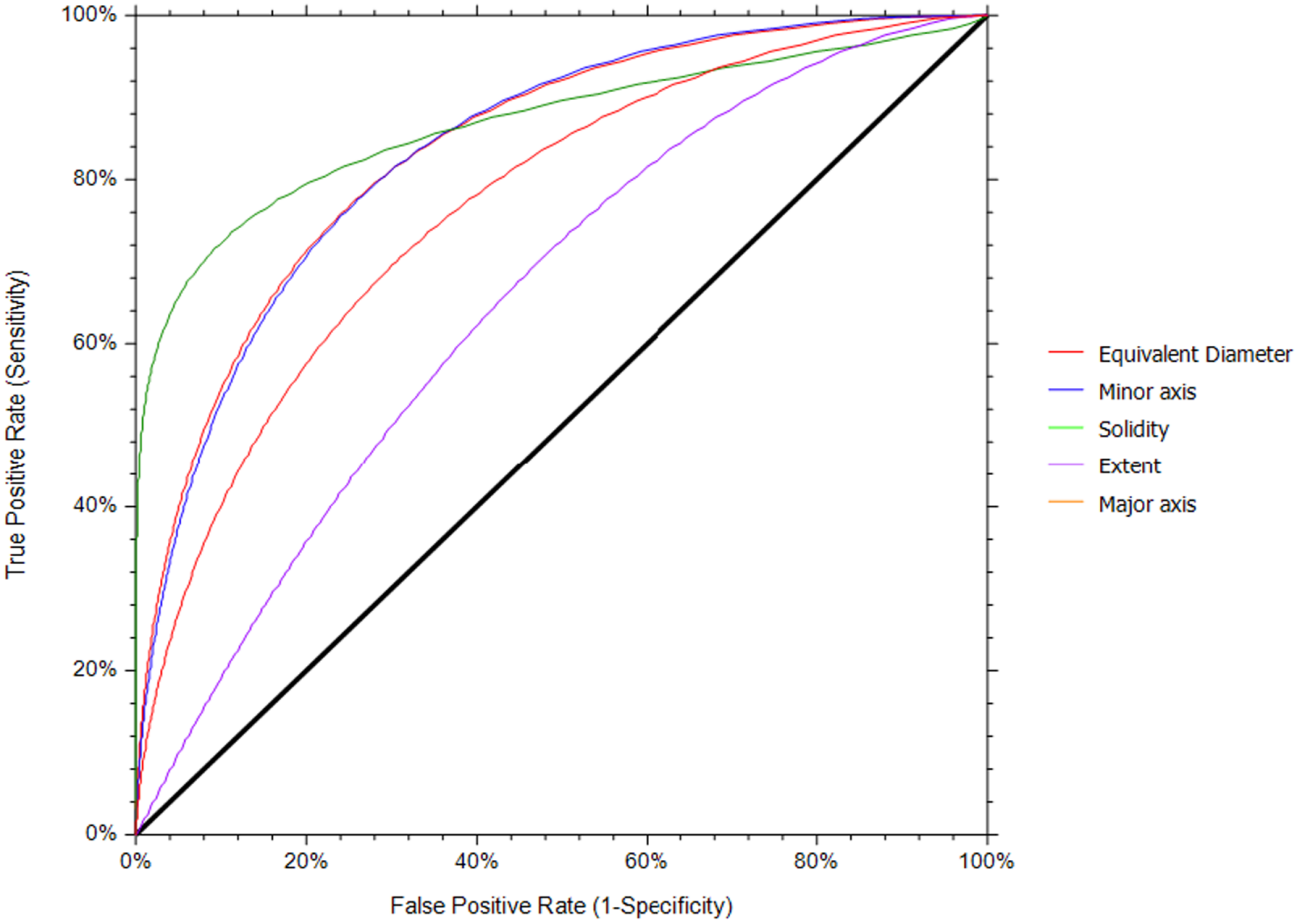
Receiver operating characteristic curve for the morphological features.

**Fig 8:**
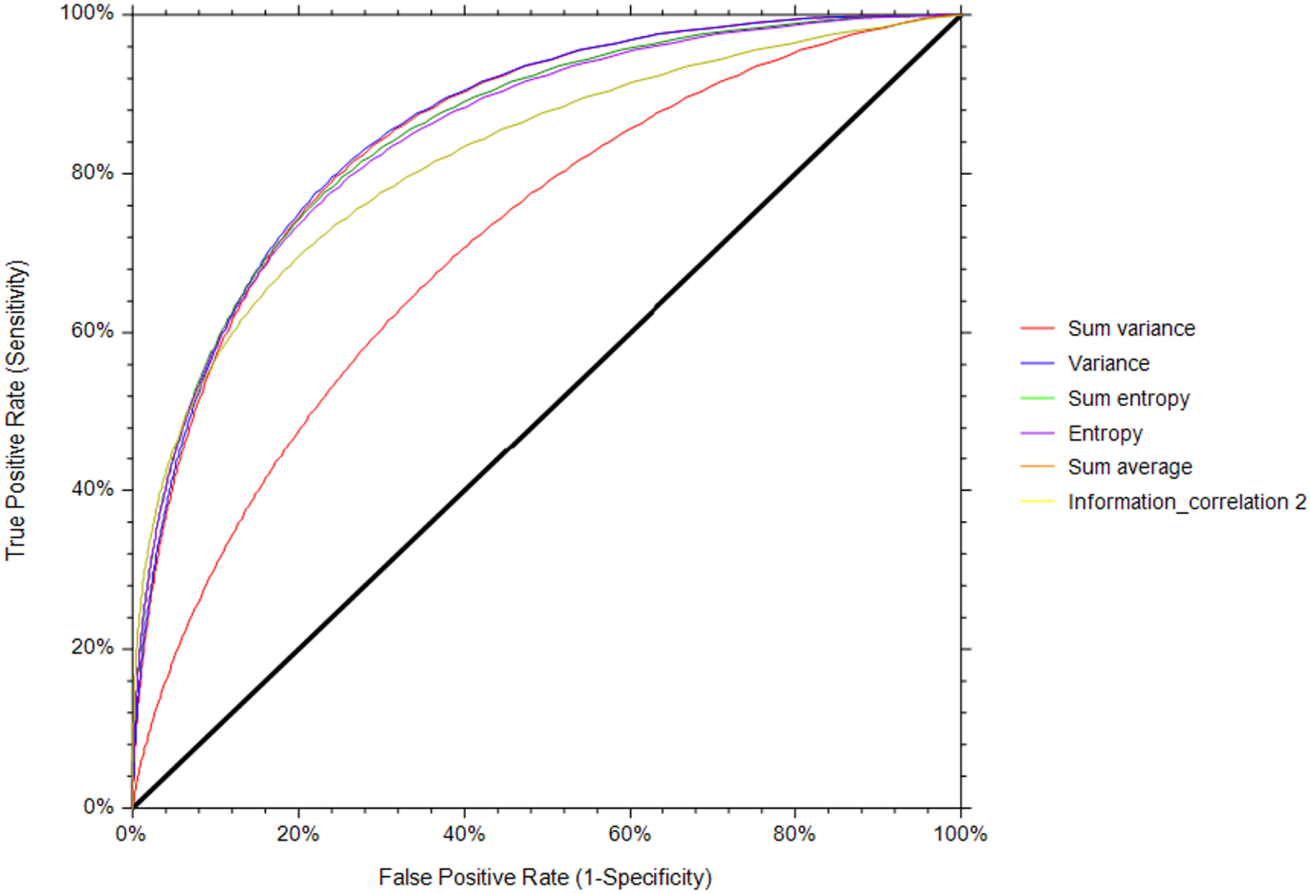
Receiver operating characteristic curve for the textural features.

The most relevant features of colony morphology and textures determined by Fisher scores using training data (40) were involved in the classification of the iPSC colony quality. On the basis of the Fisher scores, the features with the lowest score were not considered as the most relevant for quality estimation in this study. The Fisher score of each feature of colony morphology and textures for discriminating the quality is presented in S2 and S3 Tables, respectively. Only features that had a Fisher score higher than a certain threshold (e.g., 0.450) were kept, whereas others that showed a less discriminant effect on the classifier were removed. Under the colony morphology feature, the equivalent diameter, minor axis, major axis, solidity, and extent were selected as being the most relevant features, showing higher Fisher scores than the other features. Under textures, the sum variance, variance, sum entropy, entropy, sum average, and information measures of correlation_2 were selected as the most relevant features. The ranges of values of these features for the healthy colonies were higher than those for the unhealthy colonies are depicted in Fig 6. The potential of each individual morphological and textural feature in distinguishing the iPSC colonies was examined from the area under the curve (AUC), using receiver operating characteristic curve analysis (NCSS 11 Statistical Software, Kaysville, UT, USA). The performance of each individual morphological and textural feature (estimated through AUC values) in distinguishing the colonies is summarized in Table 1. Among the morphological features, solidity outperformed the rest with an AUC value of 0.878 ± 0.03 and a confidence interval (CI) of 0.747–0.933 (Fig 7). Among the textures, variance (AUC = 0.859 ± 0.03, CI = 0.741–0.926) slightly outperformed the sum entropy and sum variance in distinguishing the colonies (Fig 8).

**Table 1:** Performance validation of each individual feature, using the area under the curve (AUC), corresponding standard error (SE), and 95% confidence interval.

Features	AUC	SE	CI (Lower-Upper)
**Morphology**			
Solidity	0.8780	0.0378	(0.747–0.933)
Extent	0.7137	0.049	(0.603–0.766)
Major axis	0.7673	0.048	(0.628–0.859)
Equivalent_diameter	0.8395	0.040	(0.716–0.912)
Minimum axis	0.8377	0.041	(0.713–0.911)
**Textures**			
Entropy	0.8505	0.0392	(0.729–0.919)
Variance	0.8594	0.8594	(0.741–0.926)
Sum average	0.7129	0.0528	(0.560–0.815)
Sum entropy	0.8542	0.387	(0.735–0.922)
Sum variance	0.8567	0.384	(0.738–0.924)
Information correlation_2	0.8210	0.0431	(0.6927–0.8993)

**Fig 9:**
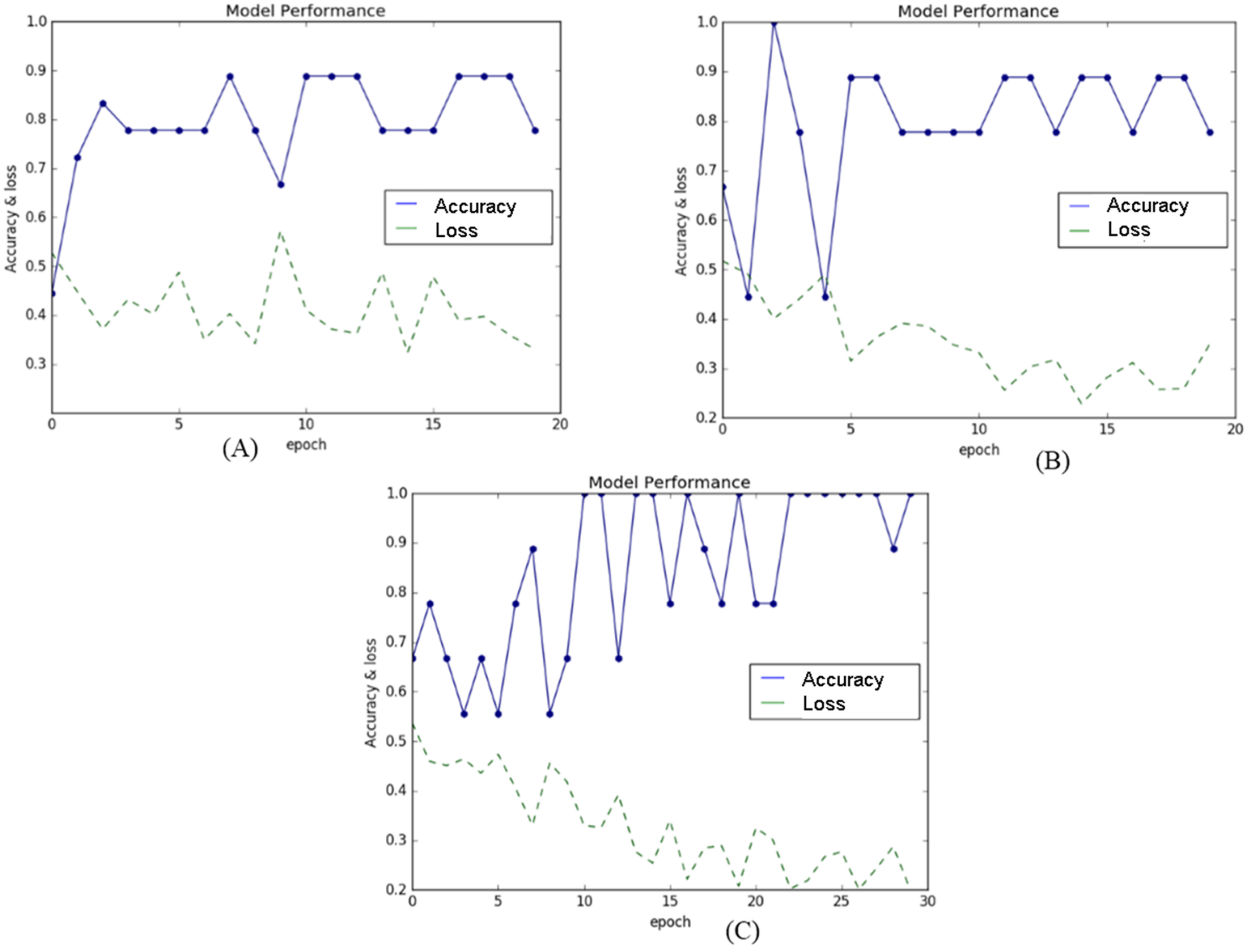
Performance of the V-CNN model with regard to accuracy and loss. The model’s performance in classifying the colonies was based on (A) morphological, (B) textural, and (C) combined features.

In order to analyze the classification performance, the selected features were inserted into the V-CNN model to distinguish the healthy and unhealthy colonies of iPSCs. Since feature vectors cannot be entered directly into the CNN network, we added a transfer function from the feature vectors (11) to the virtual image at the front of the CNN organization. We found that (28 × 28) size of the virtual image was optimum by experiment to produce better results in this study. This was then entered through a stack of two-dimensional convolutional layers of 32 filters with convolution kernel sizes of 3 × 3 throughout the operation of the network. The training process of the model was performed using a labeled dataset of input feature vectors. The testing size of 0.33 and random_state with seed value of 9 was observed to produce best performance of the proposed model. The total dataset was then divided into 60 for training and 30 for testing the model. The V-CNN model had a higher capacity in classifying the quality of colonies, as indicated by its high accuracy and low loss values (Fig 9). The estimated loss values for the morphological, textural, and combined features were low (0.209, 0.285, and 0.202, respectively), implying the behavior of the model after 20 iterations of optimization. In addition, the performance of the V-CNN model was further compared with that of the SVM classifier, using the radial basic function kernel. Quadratic programming was applied to optimize the parameters of the SVM model and the program used in the experiments was implemented using Scikit-learn toolkit [31]. The hyper-parameters *γ* that control the capacity of the kernel and *C*, the regularization parameter [34, 35] were determined by using cross-validation. The combination of parameters were observed to produce better results at *γ* = 2 and *C* = 1.

The performance of the V-CNN model in determining the quality of iPSC colonies on the basis of various feature sets is presented in Table 2. The accuracy of the morphological features in assessing the colony quality was slightly higher than that of the textural features. In addition, with the V-CNN model, the accuracy of the morphological (95.5%), textural (91.0%), and combined (93.2%) features in determining the quality of colonies was higher than that of those features (86.7%, 83.3%, and 83.4%, respectively) used in the SVM classifier. Furthermore, to validate the performance of the proposed model, precision, recall, and *F*-measures were used [6]. Precision demonstrates the number of positive predictions divided by the total number of positive class values predicted, and is defined as

$$\text{Pr}ecision=\frac{\sum_{i=1}^{c}\left(TP_{i}^{}\right)}{\sum_{i=1}^{c}(TP_{i}+FP_{i})}$$ (12)

**Table 2:** Performance of the proposed V-CNN model and SVM classifier in classifying colonies on the basis of morphological, textural, and combined features.

Features	Accuracy (%)	Precision (%)	Recall (%)	F-measure (%)
**V-CNN model**				
Morphology	95.5	93.3	93.1	93.1
Textures	91.0	87.7	87.0	87.3
Combined	93.3	89.9	89.7	89.8
**SVM classifier**				
Morphology	86.7	87.5	87.3	86.0
Textures	83.3	85.3	83.4	83.5
Combined	83.4	84.5	82.0	82.5

Recall indicates the number of positive predictions divided by the number of positive class values in the test data, and is defined as

$$\text{Re}call=\frac{\sum_{i=1}^{c}\left(TP_{i}^{}\right)}{\sum_{i=1}^{c}(TP_{i}+FN_{i})}$$ (13)

*F*-measure conveys the weighted harmonic mean of the precision and recall, and is defined as

$$F-measure=\frac{2\times \;\text{Pr}ecision\;\times \;\text{Re}call}{\text{Pr}ecision\;+\;\text{Re}call}$$ (14)

where *c* is the number of classes, and *TP*, *FP*, and *FN* represent the number of true positives, false positives, and false negatives, respectively. *TP* indicates when the model predicts the *i*^*th*^ class label as “(healthy colony)” and the *i*^*th*^ ground truth class label is likewise “(healthy colony).” *FP* indicates when the model predicts the *i*^*th*^ class label as “(healthy colony)” but the *i*^*th*^ ground truth class label is “(unhealthy colony).” *FN* indicates when the model predicts the *i*^*th*^ class label as “(unhealthy colony)” but the *i*^*th*^ ground truth class label is “(healthy colony).”

The precision, recall, and *F*-measure values generated in the current study with the V-CNN model were high that indicated the fewer numbers of false positives and false negatives than those generated by the competing SVM classifier (Table 2). Furthermore, the reliability and generalization of the proposed V-CNN model were investigated using a five-fold cross-validation method [30, 31], which functions by partitioning the datasets into *k* parts (*k* = 5). The partitioned data are represented as a fold. The method was trained on *k*–1 folds with one held back, and tested on the held back fold. This was continued five times separately, applying different members of the training and testing data that possess compositions different from those of the other experiment. The mean value of these five different compositions of classification performance was evaluated and considered as the overall accuracy of the model. The experimental results of the five-fold cross-validation of the performance of the V-CNN and SVM classifiers in determining the quality of iPSC colonies on the basis of various feature sets are presented in Table 3. The overall accuracy results using five-fold cross-validation for the V-CNN were much higher than those of the SVM, which was more than 10%. Similarly, five-fold cross-validation of the performance of the two tested models in evaluating the precision, recall, and *F*-measures produced high values in the range of 85–89% for V-CNN and very low values in the range of 68–83% for SVM, indicating the robustness and effectiveness of the proposed V-CNN approach in determining the quality of colonies.

**Table 3:** Five-fold cross-validation of the performance of the proposed V-CNN model and SVM classifier in classifying colonies on the basis of their morphological, textural, and combined features.

Features	Accuracy	Precision	Recall	F-measure
**V-CNN model**				
Morphological	92.4	89.9	89.1	89.6
Textures	90.2	85.3	85.1	85.4
Combined	91.6	86.6	86.4	86.6
**SVM classifier**				
Morphological	75.2	77.2	70.4	74.7
Textures	77.3	75.8	77.1	77.0
Combined	77.0	83.0	68.4	76.9

## Discussion

This study has proposed a new automatic system that interfaces image analysis methods with the V-CNN model for the segmentation and classification of phase contrast microscopy images, using the morphological and textural features of iPSC colonies. To our best knowledge, this is the first study to have designed a vector-based deep CNN for classification of iPSC colony quality. The motivation for implementing the V-CNN in this study was to investigate the suitability of this classifier model in accomplishing the classification of feature vectors of healthy and unhealthy colonies, which is the main contribution of this study. The V-CNN model classifier had a higher discriminant ability with colony morphologies than with colony textures. The model revealed highly acceptable classification accuracy (95.5%) compared with the SVM classifier (86.7%) that was implemented for similar feature vectors in this study. The overall accuracy using five-fold cross-validation with V-CNN and SVM was <90% and 75–77%, respectively. Furthermore, the precision, recall, and *F*-measure values of the V-CNN model were much higher than those of the SVM classifier. The differences in performance could be due to the transfer of the prior knowledge of feature maps of the V-CNN model across the stacked layers of the network capable of minimizing the classification error. Furthermore, the local shared weight capacity of the neurons drastically reduced the network complexity and number of parameters, thus contributing to the robustness of the performance in classifying the stem cell colonies.

There are several machine learning techniques that are preferred for training algorithms to classify the colonies of stem cells [7–15]. However, unstable classification performances have been demonstrated on colonies that exhibit deformable and changeable morphologies. In other studies using *k*-nearest neighbor classifier, multiclass quality evaluations of iPSCs based on local features of the colony reportedly had the highest classification accuracy of 62.4% [13,14]. Other detection methods based on the colony morphology of the stem cells, computed on overlapping blocks of images, revealed moderate accuracy (80%) by means of the linear SVM classifier [36]. A method developed for embryonic stem cell colony segmentation and tracking, using dynamic and morphological features on time data by various machine learning algorithms, revealed both low and high accuracies [8]. However, our current study with deep V-CNN executing batch-based feature vectors of colony morphology has reduced the computational complexity and produced a stable classification performance with an accuracy of 95.5% in discriminating the colonies. In addition, the cross-validated accuracy of the V-CNN classifier (92.4%), as evaluated on the basis of morphological features, was much higher than that of the SVM model (75.2%) in this study. The difference between these results is reasonable, because the proposed system with V-CNN architecture has the advantage of a broader integrated structural complexity to effectively handle a certain degree of variations of features of stem cells and hence produce excellent performance in classifying the colonies, unlike other methods.

Several recent studies have demonstrated the highly significant contributions of CNN toward microscopic cell image segmentation compared with other conventional methods [19, 37]. A deep CNN for bacterial colony segmentation demonstrated its superior performance over the SVM method, similar to our study [37]. Bacterial colony enumeration using CNN obtained improved precision and recall values that were almost similar to those obtained in the current study [19]. However, these aforementioned studies used CNN for the segmentation of image objects, whereas our current study incorporated CNN for the classification of feature vectors of images, and hence the results are strictly speaking not directly comparable. Most of the previous studies considered colony morphology to be the most important criterion for estimating colony categories [5, 8, 38]. In the study by Zahedi et al. [8], area was the best individual feature for colony discrimination. Contrary to that study, another report found the shape-based solidity feature to have the strongest ability in classifying stem cells, similar to what was found in our current study [7]. However, as the clinical target, feature descriptors, and classifier models of those studies were different from ours, the studies might not be directly comparable. The limitation of our study was the small number of training data used to build the classifier model. Further studies with a much larger number of training data, with various objectives (10×, 20×, and 40×) of iPSC colony images as well as live cell imaging evaluation, should be used to evaluate the performance of the proposed V-CNN model. Furthermore, the present model analyzed gray-level co-occurrence matrix-based texture features for quality determination. In future, the usefulness of other textural features, such as discrete wavelet and geometric moment based analysis, could be considered to enhance and generalize the proposed model.

In conclusion, our newly proposed framework of interfacing image processing methods with the V-CNN model produced encouraging results in determining the iPSC colony quality. Although the CNN has been applied before for microscopic cell image segmentation, this is the first implementation for input feature vector classification of colony quality. The suitability of the V-CNN model for addressing the classification task has been successfully shown, revealing it to have higher classification accuracy than that of the competitive SVM classifier. The proposed V-CNN-based colony identification system has been experimentally tested and cross-validated to be the most optimal model. Additionally, the proposed approach does not require much computational resources, and reduces on architectural and computational complexities, and thus it can be implemented as a valuable tracking technique in a real-time classification system. Overall, our experimental results indicated that the proposed deep V-CNN approach can allow the accurate, rapid detection of colony quality, outperforms the state-of-the-arts, and thus it can be a promising decision support model for clinical applications.

## Supporting information

S1 Table Induced pluripotent stem cells colony morphological features and their definitions. (DOC)

S2 Table Fisher scores of morphology features of induced pluripotent stem cell colonies. (DOC)

S3 Table Fisher scores of textural features of induced pluripotent stem cell colonies. (DOC)

S1 Fig Image of healthy induced pluripotent stem cells expressed by stem cell markers: A) TRA-1-60 and B) TRA-1-81 (A-B: scale bar 100μ). (TIF)
