# Supplementary material for: Deep vector-based convolutional neural network approach for automatic recognition of colonies of induced pluripotent stem cells
Source: PLoS One. 2017 Dec 27;12(12):e0189974. doi: 10.1371/journal.pone.0189974 (PMC5744970; doi:10.1371/journal.pone.0189974)
Supplement: S1 Table — (DOC) [file pone.0189974.s001.doc]

**S1 Table. Induced pluripotent stem cells colony morphological features and their definitions**

| **Morphological features** | **Definitions** |
| --- | --- |
| Area | Counting all pixels included in the object. |
| Perimeter | Total length of the object boundary |
| Centroid | The location of the center of the colony |
| Equivalent diameter | The diameter of a circle with the same area as the colony region |
| Eccentricity | The ratio of distance between the foci of the fitted ellipse and the length of it major axis |
| Solidity | The ratio of the number of pixels in colony over the number of pixels in convex hull of colony. |
| Major axis | The length of the major axis of the fitted ellipse |
| Minor axis | The length of the minor axis of the fitted ellipse |
| Extent | The ratio of the number of pixels in colony over the number of pixels in bounding box of colony. |
| Orientation | The angle between the x-axis and the major axis of the fitted ellipse that has the same second-moments as the colony |
