# Supplementary material for: Deep vector-based convolutional neural network approach for automatic recognition of colonies of induced pluripotent stem cells
Source: PLoS One. 2017 Dec 27;12(12):e0189974. doi: 10.1371/journal.pone.0189974 (PMC5744970; doi:10.1371/journal.pone.0189974)
Supplement: S2 Table — (DOC) [file pone.0189974.s002.doc]

**S2 Table. Fisher scores of morphology features of induced pluripotent stem cell colonies**

| Morphology  Features | Fisher score | 95% confidence interval | |
| --- | --- | --- | --- |
|  |  | Lower bound | Upper bound |
| Extent | **0.8633** | 0.679 | 1.051 |
| Solidity | **0.7137** | 0.534 | 0.923 |
| Area | 0.1423 | 0.051 | 0.332 |
| Perimeter | 0.2132 | 0.023 | 0.401 |
| Centroid | 0.1329 | 0.060 | 0.321 |
| Diameter | **0.5127** | 0.320 | 0.711 |
| Eccentricity | 0.1691 | 0.025 | 0.361 |
| Major axis | **0.4586** | 0.271 | 0.654 |
| Minor axis | **0.4966** | 0.310 | 0.691 |
| Orientation | 0.2763 | 0.091 | 0.443 |
