# Supplementary material for: Deep vector-based convolutional neural network approach for automatic recognition of colonies of induced pluripotent stem cells
Source: PLoS One. 2017 Dec 27;12(12):e0189974. doi: 10.1371/journal.pone.0189974 (PMC5744970; doi:10.1371/journal.pone.0189974)
Supplement: S3 Table — (DOC) [file pone.0189974.s003.doc]

| Texture features | Fisher score | 95% confidence interval | |
| --- | --- | --- | --- |
|  |  | Lower bound | Upper bound |
| Energy | 0.1585 | 0.037 | 0.351 |
| Contrast | 0.2330 | 0.046 | 0.422 |
| Correlation | 0.3112 | 0.122 | 0.431 |
| Variance | **0.5881** | 0.421 | 0.780 |
| Homogeneity | 0.1569 | 0.033 | 0.354 |
| Sum average | **0.4837** | 0.362 | 0.670 |
| Sum variance | **0.5778** | 0.397 | 0.772 |
| Sum entropy | **0.5421** | 0.351 | 0.735 |
| Entropy | **0.5488** | 0.364 | 0.743 |
| Difference variance | 0.1498 | 0.049 | 0.347 |
| Difference entropy | 0.1832 | 0.012 | 0.372 |
| Information measure of correlation_1 | 0.3210 | 0.134 | 0.445 |
| Information measure of correlation_2 | **0.5211** | 0.331 | 0.712 |

**S3 Table. Fisher scores of textural features of induced pluripotent stem cell colonies**
