# Supplementary figures and images for: Deep vector-based convolutional neural network approach for automatic recognition of colonies of induced pluripotent stem cells
Source: PLoS One. 2017 Dec 27;12(12):e0189974. doi: 10.1371/journal.pone.0189974 (PMC5744970; doi:10.1371/journal.pone.0189974)

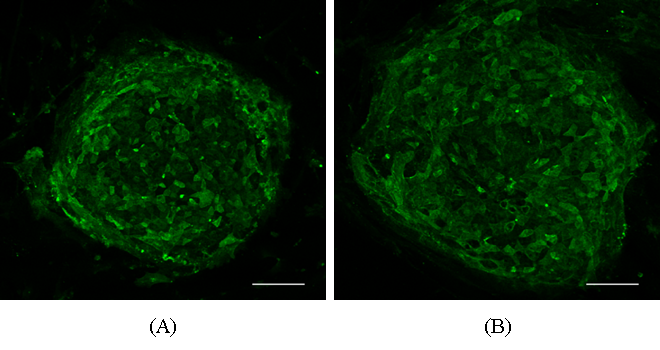

Supplement: S1 Fig — (TIF) [file pone.0189974.s004.tif]
